# An in-depth analysis of factors influencing small-scale cattle farmers’ participation in livestock markets in Western Province of Zambia: navigating challenges and barriers

**DOI:** 10.3389/fvets.2024.1397000

**Published:** 2024-11-19

**Authors:** Chisoni Mumba, Bertha Kasanga, Chisanga T. Mwamba, Timothy Sichilima, Enock Siankwilimba, Doreen C. Sitali, Joshua Munkombwe, Reuben Banda, John Bwalya Muma

**Affiliations:** ^1^Department of Disease Control, School of Veterinary Medicine, University of Zambia, Lusaka, Zambia; ^2^District Veterinary Office, Ministry of Fisheries and Livestock, Lusaka, Zambia; ^3^Musika Development Initiatives, Lusaka, Zambia; ^4^Department of Health Promotion, School of Public Health, University of Zambia, Lusaka, Zambia

**Keywords:** Livestock markets, socioeconomic factors, cultural factors, smallholder-cattle-farmer, Zambia

## Abstract

**Introductions:**

The low participation of small-scale farmers in livestock markets in sub-Saharan Africa, including Zambia, is a major inhibitor to private sector investment in the livestock subsector. Despite the immense potential of the livestock industry to bolster the economic development of countries in this region, several socioeconomic, environmental, and cultural factors contribute to this hindrance.

**Methods:**

This study was conducted in the Western Province of Zambia and adopted a qualitative research approach to understand the challenges and barriers affecting livestock markets among smallholder cattle farmers. In-depth interviews were conducted with 23 key informants and focus group discussions involving key actors in the dairy and beef value chains. Thematic analysis was employed to analyze the gathered data.

**Results:**

Four themes, including socioeconomic, cultural, market dynamics, and policy and regulatory factors, emerged from this analysis. The sub-themes for socioeconomic factors included access to capital, infrastructure challenges, limited technical knowledge, and inadequate veterinary services. Cultural factors included traditional practices, social norms, and perceptions of livestock, and subthemes for market dynamics included price volatility, lack of market information, and middlemen exploitation. The sub-themes for policy and regulatory factors included policy inconsistencies, land tenure issues, and inadequate government support.

**Conclusion:**

By identifying and understanding these factors, policymakers and stakeholders can develop effective and sustainable targeted interventions and policies to encourage smallholder cattle farmers’ participation in the livestock sector.

## 1 Introduction

Livestock markets play a crucial role in enhancing livestock production, serving as a key driver for both economic and social benefits (1). These markets exert both pull and push effects on livestock production, incentivizing farmers to improve productivity. The prospect of market participation motivates farmers to adopt modern livestock management techniques, such as disease control, improved feed, and better housing, all in an effort to meet market standards (2).

However, in the Western Province of Zambia, there is a notable disconnect between the theoretical incentives provided by markets and the actual outcomes, particularly concerning cattle population growth and market participation. While livestock markets generally stimulate productivity and encourage farmers to enhance the quality of their livestock, the situation in this region indicates that market interventions, both in the beef and dairy industries, have not significantly increased participation in livestock markets.

A study conducted in Ethiopia revealed a similar phenomenon, where despite the important role of market participation in improving livelihoods and household income, many livestock producers did not fully engage in livestock markets (2). Several studies have investigated the factors affecting livestock farmers’ participation in markets (1, 2). However, these studies primarily relied on quantitative data collection and analysis approaches, which failed to fully uncover the socio-cultural context of the factors affecting participation. This study aims to fill that gap by using qualitative data collection and analysis techniques. This holistic approach will help understand the various factors influencing livestock farmers’ participation in markets, using the Western Province of Zambia as a case study.

Cattle production in the Western Province of Zambia is integral to the region’s socioeconomic landscape. Notably, there has been a fluctuation in the cattle population from the second highest to the fourth highest in the country between 2015 and 2022, indicating a dynamic scenario (3). Despite systemic challenges leading to a decrease in overall cattle herd numbers, the Western Province of Zambia remains a significant player in the national livestock sector (4). Endowed with vast grazing land and water bodies, Western Province has a huge potential for livestock production.

Interestingly, only a third of households in the Western Province are involved in milk production, raising questions about cattle use dynamics and the region’s economic potential compared to the Southern Province, where over two-thirds are engaged in milk production (3). Mumba et al. (5) and World Bank (6) attribute this disparity to low cattle productivity, poor herd health, poor access to dairy markets, and entrenched traditional practices, posing a threat to both the livestock subsector’s viability and the livelihoods of dependent smallholder farmers. Other studies have attributed these cyclic challenges to low entrepreneurial acumen entrenched in traditional beliefs and norms, which makes it hard to let go of smallholder farmers (4, 6, 7).

These inefficiencies prompted previous stakeholder support for beef and dairy interventions in Southern and Western provinces. However, observations over time reveal a significant contrast in the success of beef and dairy interventions, with the Southern Province outperforming the Western Province (8, 9). Therefore, this study aims to analyze the various factors that affect the participation of small-scale cattle farmers in livestock markets in Western Province of Zambia. By identifying challenges and barriers these farmers face, the research seeks to provide insights for navigating these obstacles, ultimately improving their access to livestock markets.

## 2 Materials and methods

### 2.1 Description of study sites

**Figure 1:**
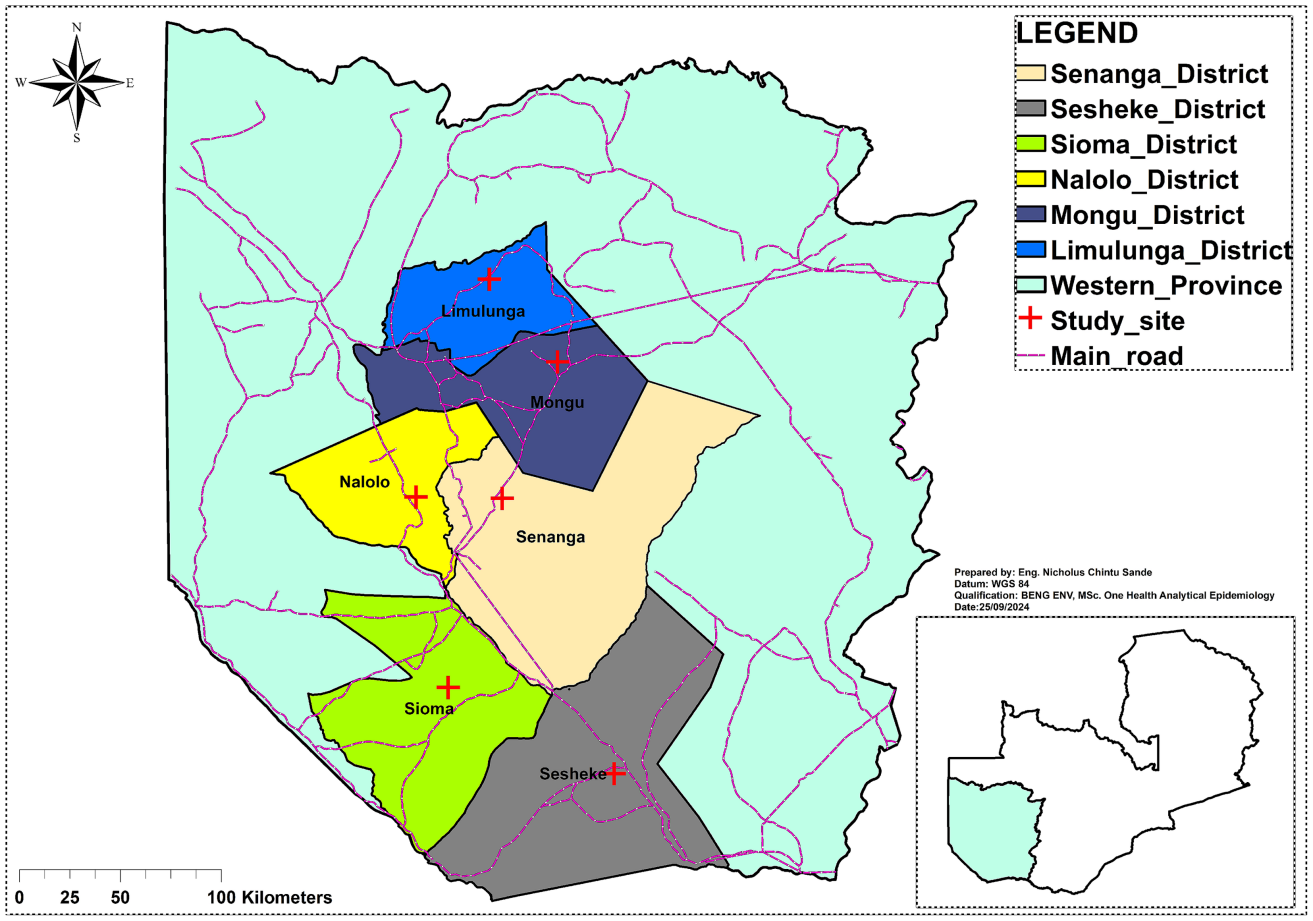
Map of Western Province showing study sites (developed by authors).

The study focused on the Western Province, specifically targeting key value chain actors in the dairy and beef sectors. The province was chosen because of the plummeting cattle numbers from being the second highest to the fourth highest in the country between 2015 and 2022 (3). Data was collected in six districts: Senanga, Sesheke, Sioma, Nalolo, Mongu, and Limulunga as shown on the map in Figure 1. The selection of these districts was based on the presence of beef processors and Milk Collection Centres (MCCs), ensuring representation from critical points in the value chains.

Zambia is classified into three main agroecological zones according to pedological characteristics, climatic factors, rainfall patterns and common agricultural practices. The three ecological zones extend from the west to the east of the country with Agroecological Zone I in the South, Agroecological Zone II in the north of Agro-ecological Zone I and Agro-ecological Zone III further to the north covering parts of the North-western, Northern, Luapula, and Muchinga Provinces with the highest rainfall.^1^

Western Province falls primarily within agroecological Zone I and partially in agroecological Zone II of Zambia. The province has low rainfall, typically receiving between 600 mm to 800 mm of rainfall annually. This classifies much of the area as semi-arid. The primary zone within Western Province is known as the Barotse Floodplain or the Zambezi Floodplain, where flooding during the rainy season makes the land suitable for fishing and seasonal agriculture. The region’s agroecology is well-suited for livestock farming (especially cattle) and limited crop farming due to the sandy soils and low fertility. Western Province is one of the four leading regions for cattle rearing in Zambia, standing at Number four from Southern, Eastern and Central provinces with a cattle population of 429,142 (3, 10). Cattle ownership is a critical part of the socio-economic fabric in rural areas, serving not only as a source of wealth but also for draft power, milk, and manure (10). Other livestock include goats, poultry, and pigs, though cattle dominate in numbers and importance. Livestock diseases, particularly foot-and-mouth disease (FMD) and Contagious Bovine Pleural Pneumonia (CBPP), are major challenges in this region due to the transboundary nature of livestock movement with neighboring countries like Namibia and Angola (11).

### 2.2 Study design

The research adopted a qualitative case study design. The design was appropriate for building an in-depth understanding of factors influencing small-scale cattle farmers’ participation in livestock markets by relying on multiple data sources (12, 13). The Western Province of Zambia was considered as a case represented by the six districts selected based on presence of dairy and beef markets. The chosen methods included in-depth interviews and focus group discussions. This integrated data collection technique aimed to capture insights from key actors involved in the dairy and beef value chains.

#### 2.2.1 Sample size estimation for key informant interviews and focus group meetings

The sample size for key informant interviews was 23, and five (5) for focus group discussions (FGD). The sample size (N) was determined after reaching a data saturation point for key informant interviews and focus group discussions. Strauss and Corbin (14) state that researchers should collect data until “no new or relevant data” emerges regarding a category. Therefore, interviews were conducted with different categories of informants in the dairy and beef value chains following a line of “theoretical sampling” to continuously construct and refine the hypothesis in terms of reference. The method of determining saturation in data collection was an iterative process for each interview, which involved recruiting key informants or focus groups, interviewing them, and analyzing the data.

#### 2.2.2 Sampling technique for key informant interviews

A total of 23 key informant interviews were purposively sampled based on their involvement with beef and dairy cattle farmers in the districts. Table 1 indicates the key informants in the small-scale dairy and beef value chain that were interviewed. Key informants were interviewed from their offices. Efforts were made to interview all those at the provincial office on the same day in different offices to avoid discussion before the interviews. Appointments were made via the phone, and physical interviews with each key informant were conducted. All interviews were recorded verbatim. Verbal consent to record the interviews was sought. Interviews were conducted in the English language.

**Table 1:** List of key informants.

Key informant	Number
Provincial Veterinary Officer	1
Provincial Fisheries and Livestock Coordinator	1
District Fisheries and Livestock Coordinator	2
District Veterinary Officer	2
Senior Veterinary Officer	1
Senior Livestock Production Officer	1
Livestock Technicians	2
Veterinary Assistants	1
Beef Processors	1
Dairy Processor	1
Dairy Farmers (Pure breeds)	2
Cattle Producer Association	1
Transporter	1
Trader	1
Politicians (mayor and member of parliament)	2
Service Providers (extension services)	2
Academician (Lecturer)	1
Total	23

#### 2.2.3 Sampling technique for focus group discussions

A total of five focus group discussions were conducted. The focus group discussions with farmers in the smallholder dairy and beef value chain included Nalolo Dairy Cooperative, Limulunga Dairy Cooperative, Mongu Dairy Cooperative, Senanga Dairy Cooperative, and the Barotse Royal Establishment. Apart from the one conducted with the Barotse Royal Establishment (BRE), all focus group discussions involved a mixture of men and women in dairy and beef value chains. The minimum number of participants in each group was 6, with a maximum of 13. Focus group discussions from Nalolo and Senanga were conducted in a local language (Silozi) with the help of a livestock and veterinary assistant as an interpreter.

#### 2.2.4 Preparation for focus group discussions and selection of participants

The research team planned the organization of the meetings over a three-week period, which is important to mobilize stakeholders and successfully carry out focus group discussions, as highlighted by Andersen and Richardson (15). During this period, logistics were put in place, and the checklist or agenda for the focus group discussions was developed as guided by Rich et al. (16). Since Western Province is 600 km from Lusaka Province, where the researchers were based, the gatekeepers (contact persons with the stakeholders) played a key role in inviting the stakeholders by delivering invitation letters and the agenda for the meetings as guided by Mumba et al. (5). Gatekeepers represent the link between the facilitators of focus group meetings and stakeholders, thus playing a brokering role and controlling access to the community (17). The selection of participants for the focus group discussions was left to the chairperson of the dairy cooperatives, with the guidance to include active members. Participants in the focus group discussions were not involved in any survey or discussion by the facilitators to prevent them from coming up with preconceived ideas for the meeting.

### 2.3 Data management and analysis

Audio files that were in the local language (Silozi) were translated and interpreted into English. The audio files for both key informants and focus group discussions were then transcribed into computer files. After reading and re-reading the narratives, the data were coded manually by two researchers. Initially, broad coding was done using significant themes derived from the topic guides in the checklist. This was followed by further coding of the major themes into sub-themes. Framework matrices were then formulated by identifying key quotes under each sub-theme and summarizing the narratives under each major theme. Framework matrices were used to cross-check narratives from key informants with focus group participants to identify divergent or supporting views. Raw narratives have been provided in the results. Identities for companies and officials that had interventions in the beef and dairy industries in the study areas were withheld and only replaced by anonomity in the narratives provided in the results section.

### 2.4 Conceptual framework

**Figure 2:**
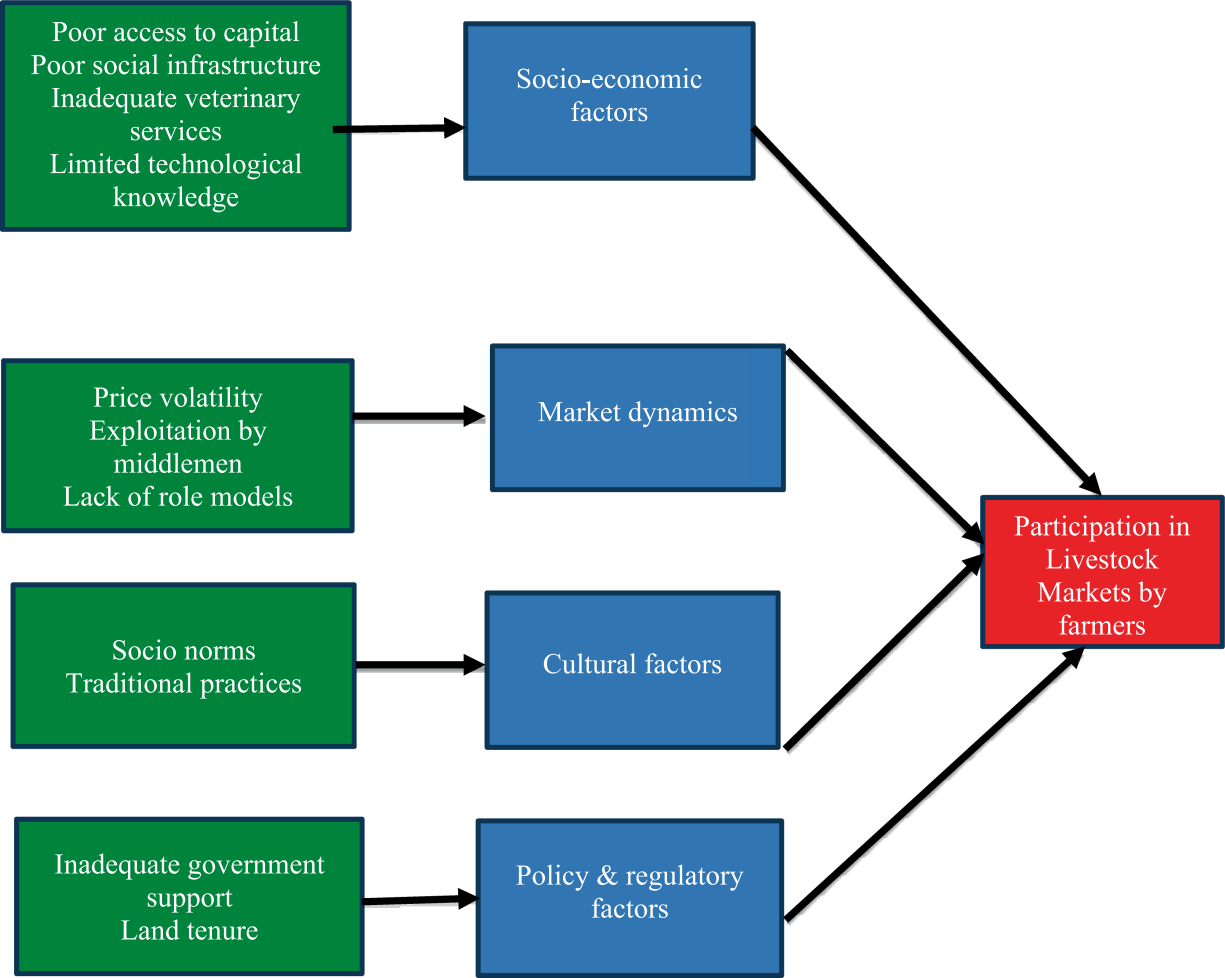
Conceptual framework for participation of farmers in livestock markets.

Figure 2 summarizes the conceptual framework of the study. Four themes, including socioeconomic, cultural, market dynamics, and policy and regulatory factors, emerged from this qualitative data analysis. The sub-themes for socioeconomic factors included access to capital, infrastructure challenges, limited technical knowledge, and inadequate veterinary services. Cultural factors included traditional practices, social norms, and perceptions of livestock, and subthemes for market dynamics included price volatility, lack of market information, and middlemen exploitation. The sub-themes for policy and regulatory factors included policy inconsistencies, land tenure issues, and inadequate government support. The results have been presented and discusses within the main themes.

## 3 Results and discussion

This section presents and discusses results under four themes: socioeconomic, cultural, market dynamics, and policy and regulatory factors that emerged from the thematic analysis. A total of 19 sub-themes were derived from the main themes, and these sub-themes include:

### 3.1 Absence of commercial farmers in Western Province

The key informants reported that, unlike the Southern and Central Provinces of Zambia, there were no commercial livestock farmers in the Western Province. This observation suggests a regional disparity in the scale and nature of livestock farming. Western Province does not exhibit a notable presence of large-scale or commercial livestock operations as seen in other provinces. Understanding these regional variations is crucial for tailoring effective interventions and policies that align with the specific dynamics and challenges faced by the livestock industry in Western Province. A key informant, who is quoted below, stated that:

*“I would say that the farmers in Western Province do not have role models. So there is no such farmer where the farmers can go and get ideas on how to run the dairy farming… they can adapt very fast…”*

This absence of commercial farmers is attributed, among other factors, to the traditional land tenure system (18). The commercial farmers want title deeds to the land as this provides collateral for them to access credit facilities for investment, but the land tenure system in Western Province does not easily facilitate this compared to Southern Province. Commercial livestock farmers serve as role models where small-scale farmers can learn animal husbandry skills. Creating platforms to showcase successful farmers from other provinces, providing mentorship programs, and establishing knowledge-sharing networks can help small-scale farmers learn from successful commercial farmers. A study by Copenhagen Social (19) demonstrates that the Silverland smallholder livestock commercialization project in the Zimba district of the Southern Province in Zambia is an exemplary instance in which a commercial entity engaged smallholder farmers in a commercial partnership with the goal of transferring farm-based business innovation to the local community.

### 3.2 Lack of improved dairy breeds

The study findings indicated that farmers in the Western Province predominantly use the local Barotse breed for milk production. However, the Barotse breed’s milk production is limited, with a maximum of about 2 liters per day, due to factors such as poor nutrition and low genetic potential for milk production (8). Key informants reported that the Barotse breed typically produces 0.5–1.0 liters per day for a short lactation period of 6 months.

Efforts to enhance the Barotse breed through artificial insemination with Friesian and Jersey semen have faced challenges, including low conception rates, dropping as low as 20%. Consequently, farmers have lost confidence in artificial insemination as a viable improvement strategy. Factors contributing to the low conception rates include poor animal nutrition, inadequate handling of semen (cold chain), prevalent animal diseases, and the transhumance cattle herding system. The study also highlighted a significant deficiency in extension services, particularly in guiding the preparation and management of animals for insemination, contributing to the observed challenges in improving the Barotse breed. A key informant had this to say about improving breeds:

*“Then the artificial insemination that is being promoted by anonomity Genetic breeders and its partners, I think, in these animals is a bit chaotic. Okay, the system needs to be done properly. You know you need to isolate the female nicely to avoid disturbance by bulls, and you need a good plan of nutrition because the animals are seasonal breeders. They only give calves at a certain time of the year when the grass is okay, so you cannot inseminate anytime without some supplementary feeding, and the results have been very poor. The results have been very poor; we need to work on that. Several programs have come; we had money from anonomity to promote artificial insemination, but even then it did not work out because, okay, you need a paddock, you need to isolate animals, you need to feed them, and then they pick very nicely. So the idea was that we just buy semen from the improved breed and inseminate them. I think these are things that we need for artificial insemination to be improved. Or if it is bringing bulls, we need to work on something like that; otherwise, the program is very much needed…”*

Further to the earlier observation of the absence of commercial farmers, this could also contribute to the absence of low-grade cattle breeds in the province. Commercial farmers often serve as a source of high-value breeding stock from which small-scale farmers in proximity have the opportunity to purchase and improve their cattle herds.

### 3.3 Inadequate extension services

The study uncovered a lack of adequate government support regarding extension services in the Western Province. Government-dominated extension services face numerous challenges, particularly in the veterinary sector, where officers in many camps lack essential resources such as motorbikes and fuel. This inadequacy in extension services poses challenges for implementing interventions, particularly in clinical services, as there are limited private institutions capable of filling this gap. Extension services are crucial for farmers, playing a vital role in their participation in livestock markets. However, the study suggests that the current limitations in government-led extension services hinder the effective implementation of interventions. The involvement of the private sector in extension services is emphasized as a key factor in addressing these challenges and enhancing support for livestock farmers in the Western Province. But again, the absence of commercial farmers in the province implies little incentive for private veterinary service providers to set up enterprises to offer such extension services. The study underscores the importance of collaborative efforts between the public and private sectors to improve extension services, ultimately contributing to the development of the livestock industry in the region. A key informant is quoted saying that:

*“The terrain in Western Province and the rigidity of cattle farmers discourage private sector interventions. So investors that are looking at huge turnovers over a short to medium timeframe may fail in the market and prematurely leave the interventions…”*

Key informants highlighted that rigidity among farmers in the Western Province stems from several factors. Firstly, the practice of not treating cattle farming as a business contributes to inflexibility. Additionally, there is a lack of sensitization among farmers, and adherence to traditional (cultural) methods of animal production, where animals grow without adequate animal health management practices until market age in some cases, further reinforces this rigidity. Another factor is the resistance to adopting and paying for extension services, as farmers are accustomed to receiving these services for free. This reluctance is partly due to the assumption that all private investors are involved in grant-aided projects, which typically offer services without charge. To address these challenges, the study suggests a need for capacity building within the government extension staff or system. This capacity building should be done sustainably through a private-public partnership (PPP) approach for any livestock intervention. The incorporation of PPP aims to foster collaboration between the public and private sectors, ensuring a more effective and sustainable approach to livestock interventions in the Western Province.

### 3.4 Lack of a business mindset and skills

The study revealed that most livestock farmers in the Western Province lacked a business mindset when it comes to actively participating in livestock markets. The interventions implemented thus far have not sufficiently sensitized farmers regarding the concept of farming as a business. Consequently, livestock farmers are not making significant investments in their cattle. Livestock markets in the Western Province have faced challenges, particularly due to prolonged livestock movement bans imposed in response to diseases like Contagious Bovine Pleural Pneumonia (CBPP) (11). This has hindered farmers from accessing high-value markets and making informed investments in their livestock businesses.

Traditionally, livestock farmers in the region keep cattle for prestige, only selling animals when facing urgent financial needs, such as when requiring paying school fees for their children. This practice results in reduced cattle sales during times when financial pressures, such as school fees, are not immediate concerns, as seen during the closure of schools in March 2020 due to the COVID-19 pandemic. Consequently, farmers tend to view selling livestock more as an optional liquidity strategy than a business endeavor. The disease also impacts livestock off-takes, forcing sick animals into imposed sales (20). The beef sourced from traditional cattle farmers primarily comes from culls involving old and sick animals (20).

The lack of a business mindset and skills poses challenges for farmers to actively participate in both the beef and dairy markets. For dairy markets, in particular, sensitization and training are identified as crucial components to encourage farmer participation even after the introduction of market interventions. The study underscores the need for comprehensive education and training efforts to shift livestock farmers’ mindset toward viewing their activities as viable business enterprises. A key informant stated that:

*“People also need to be taught to understand that livestock is a business; you cannot come from nowhere; just embark on livestock farming and achieve what you have been dreaming about. But then people here have inherited animals; they have become farmers through inheritance with no capital injection. So you find that if I have inherited 40 animals from an uncle, I will be told two animals have died. So what? It still does not count for you because you have not spent anything. So we need to teach these people that you could have inherited these animals fine, but we need a mindset shift and technology transfer…”*

### 3.5 Poor road infrastructure

The study highlighted significant challenges in the road infrastructure of the Western Province. Apart from the Mongu-Kalabo road and the main Lusaka-Mongu Road, which is also in poor condition between Nkeyema and Luampa districts, more than 90% of the feeder road network was characterized as poor. These feeder roads predominantly feature sandy terrain, limiting access to rural areas where cattle farming activities primarily take place in the province. Roads such as Kaoma-Lukulu-Mitete, Sioma-Shangombo, Sesheke-Kazungula, and Kaoma-Kasempa were reported to be in a bad state, impacting participation in livestock markets and discouraging private sector investment.

The poor condition of roads not only affects the transportation of input supplies but also poses challenges for delivering milk to aggregation points. Milk collection centers, situated along main tarred roads, become difficult to reach by farmers who have to travel some distances through the sandy terrain, making popularly used bicycle transportation challenging. The Kaoma-Kasempa road, which plays a role in trading livestock with the mining towns of Solwezi and Kasumbalesa, was also reported to be in poor condition.

Poor road infrastructure was identified as a hindrance to private sector investment in livestock markets in the Western Province. This, in turn, affects the participation of livestock farmers. The study underscores the interconnectedness of markets and livestock production, emphasizing the need for improved road infrastructure to facilitate market access and enhance the overall livestock value chain in the region. The study points out that a significant number of traditional cattle farmers are situated in rural areas lacking proper feeder road infrastructure. This absence of good feeder roads poses challenges for both accessing better markets and obtaining essential inputs for the farmers. The inadequate road infrastructure limits the opportunities for these farmers to improve market access and enhance their livestock farming activities. Additionally, inadequate road conditions can potentially lead to escalated transportation expenses and impede the movement of livestock to market locations. Transport is a key enabler for the development of the dairy value chain.

### 3.6 Lack of supportive production and market infrastructure

Both participants of key informant interviews and focus group discussions indicated a lack of production and market infrastructure in Western Province (see quote):

*“Then infrastructure, both market infrastructure and production infrastructure, are nowhere to be seen, so there are no water troughs, no feeding troughs, no paddocking system, and no water supply, so animals are made to change like the weather. When it rains, they are happy, and animals need to be provided for. So based on that, we have found ourselves in a situation where we forsake what would be the most profitable industry for the province…”*

Beyond the absence of supportive production infrastructure, farmers in the Western Province are unwilling to invest in production infrastructure. This reluctance is attributed to a broader issue—farmers’ lack of a business mindset. As previously mentioned, the traditional way of keeping cattle contributes to this mindset, where cattle farming is often viewed more as a traditional practice for prestige or meeting immediate financial needs than as a business with long-term investments (10).

The combination of insufficient production infrastructure and hesitancy among farmers to invest in such infrastructure points to a broader challenge in shifting the prevailing mindset and encouraging a more business-oriented approach to livestock farming. Addressing this mindset issue is essential for promoting sustainable investments, improving productivity, and fostering the overall development of the livestock sector in Western Province. It emphasizes the importance of targeted interventions, sensitization, and capacity-building efforts to instill a business-oriented perspective among livestock farmers in the region. Therefore, if not addressed, insufficient training and capacity-building programs may continue to hinder farmers from acquiring the necessary skills for successful market engagement. Strengthening farmers’ capacity through education and training can empower them to participate more effectively in livestock markets.

### 3.7 Seasonality of milk production in transhumance cattle herding systems

The study indicates that grazing areas in Western Province are considered common property under the custodianship of traditional leaders. This communal ownership structure makes private investments challenging, as returns on public goods cannot be internalized. Consequently, this communal approach to grazing areas poses difficulties in enhancing productivity among traditional livestock farmers.

Under the transhumance cattle herding system in the region, animals move from the floodplains, where there is abundant pasture, to the upper land, where pasture is insufficient. This movement typically occurs from March to August, with animals returning to the Zambezi floodplain as water levels recede. As a result, milk production is limited from August to January. The lack of a paddocking system in the floodplain and upper land further complicates livestock management.

In the dry season, there is no water supply on the upper land, making it impractical to leave lactating cows. Consequently, farmers need to employ two cattle herders—one for the floodplain and another for the upper land. This dual herding system increases production costs, ultimately influencing milk pricing for consumers. The complexity of this system adversely affects farmers’ participation in livestock markets, highlighting the need for interventions to improve grazing management practices and make livestock farming more economically viable in Western Province. A key informant had this to say:

*“I think the major factor is seasonality, in Western Province, the animals move away from the plain, to the upland during this rainy season. Much of this upland does not have feeder roads, much of this upland, lies in sand tracks, so where the animals go, you cannot go and collect the milk. So the transhumant, the movement of cattle from the floodplain on the upland is a big factor…”*

The transhumance cattle herding system adds another layer of complexity by leading farmers to move from one dairy cooperative to another. For instance, a farmer may be a member of the Mongu dairy cooperative, but as their animals move far into the floodplains, the nearest Milk Collection Center (MCC) might be associated with the Nalolo dairy cooperative. In such situations, farmers have no option but to negotiate with the nearest MCC to deliver their milk during the period when their cattle are grazing in that specific area. This practice of shifting between cooperatives, driven by the movement of animals to grazing areas, results in a reduction in membership at the primary cooperative (e.g., Mongu dairy cooperative). The continual movement of members adversely affects the stability and operations of the primary cooperative, potentially creating challenges in maintaining consistent operations and services. The fluid nature of cooperative membership due to the transhumance system highlights the need for adaptive and flexible cooperative structures to accommodate the mobile nature of livestock farming in the region. As such, the transhumance system is part of Traditional beliefs and cultural practices that influence farmers’ decisions regarding when and how to sell their cattle and milk. It is clear that social networks and community dynamics play a role in shaping farmers’ attitudes towards market engagement.

### 3.8 Livestock ownership

The study reveals a unique challenge in Western Province, where livestock owners are primarily located in urban centers, such as Lusaka, the capital city of Zambia. Meanwhile, custodians, who manage the day-to-day activities of the animals, are the ones present in the local areas. This situation makes adopting interventions difficult, as custodians lack decision-making authority without the owner’s consent.

Custodians responsible for caring for the animals are compensated through free access to manure and milk produced by the livestock. However, they cannot make decisions about adopting interventions independently without the owner’s explicit approval. Even if suggestions or ideas for improving aspects like milk production are presented to the custodians, they may hesitate to implement them without the owner’s consent.

The precarious nature of this arrangement is highlighted by the fact that if a custodian were to adopt interventions without the owner’s permission, the owner might, upon returning, reclaim the animals and assign them to someone else. This not only disrupts the continuity of interventions but also requires extension workers to initiate discussions with a new person to implement the interventions.

This dynamic emphasizes the importance of engaging and obtaining consent from the actual owners of the livestock, who may be residing in urban areas, to ensure the successful implementation and continuity of interventions in Western Province. Addressing this challenge requires strategies that consider the involvement and cooperation of both custodians and owners in decision-making processes related to livestock management and interventions. One key informant stated that:

*“For instance, there was a farmer who was given animals by the sister who is in Lusaka, and he joined a dairy cooperative where he raised about ZMK 9000 (about US$530 then) from milk sales, but when the owner of the animals heard about the success story, she came to grab the animals and gave them to someone else. This practice makes participation in livestock markets difficult…”*

Another factor affecting participation in dairy markets in Western Province is the frequent change of handling of animals from one cattle keeper to another. In these transitions, the initial keeper, who is a member of a dairy cooperative, surrenders both milk and manure associated with the animals to the new keeper.

This practice of transferring handling of cattle introduces a level of complexity in tracking and managing dairy production within the cooperative. The change in ownership not only impacts the consistency of the milk supply but also involves the transfer of benefits, such as manure, from the initial keeper to the new one. It implies that the cooperative needs to adapt its strategies to address the fluid nature of ownership, ensuring that benefits are equitably distributed and that production records remain accurate despite these changes.

The continuous shifting of keepership adds a layer of challenge to managing dairy production and cooperative operations, emphasizing the need for adaptable and flexible systems to accommodate these dynamics and sustain effective participation in dairy markets. One key informant stated that:

*“And to make it worse, sometimes the animals, when they go, especially upland, change hands. The owners remain in the plains because animals leave earlier than people. Normally, when someone wants to use those animals for manuring his field or whatever, they give them to somebody else at that time. I think that also needs to be improved. I think they should not do that, but I think they have gotten used to, part of the year, having the animals go to somebody else…”*

Another cultural practice that may affect participation in milk production is Mafisa. A key informant described Mafisa as, ***“**Mafisa is when you have animals you give, because here, what it is is that it is challenging to keep many animals in the same place, like 500 or 600 cattle, because of the communal system of grazing. So if you have a lot of animals, you would get some of them, maybe about 10 or 20; you would give somebody else who will be looking after them for you. In the process, you give him one animal every 2 years**.”***

The cultural practice of Mafisa involves the owner of livestock entrusting another family with the care of a certain number of cattle. However, despite this arrangement, the decision-making authority remains with the original owner, and the current keeper does not have the autonomy to participate in dairy markets or make decisions about animal health management interventions. This dynamic also results in a lack of willingness to pay for extension services by the current keeper, who may not perceive the animals as their own.

The practice of Mafisa, where ownership and decision-making authority are not transferred to the family keeping the animals, poses challenges for private sector investment in extension services in Western Province. The reluctance of the current keeper to pay for services affects the returns on investment for private sector interventions, hindering the overall progress of livestock-related initiatives.

Moreover, Mafisa is identified as a cultural determinant of the spread of anthrax and Contagious Bovine Pleuro Pneumonia (CBPP) in the Western Province (21, 22). This further underscores the importance of considering cultural practices in designing and implementing interventions related to livestock development and animal health management.

The study suggests that livestock development interventions should consider the dual ownership structure, both the owner remaining in the floodplain and the urban owner, who may be outside Western Province. Establishing linkages between the urban owner and traditional leadership is identified as crucial for the smooth implementation of livestock interventions in this cultural context (5).

### 3.9 Consumption habits and beliefs

The cultural preference for sour milk as a relish and primary protein source in some parts of Western Province presents a challenge for farmers in selling their milk. Sour milk, being a crucial dietary component, is often retained for household consumption rather than being sold. This practice limits the quantity of milk available for sale, which is also the primary source of animal protein for farmers who may be unable to afford meat.

Additionally, there is a belief in some areas that the cow should not be milked for a significant period, approximately up to a year after the calf is born. This belief stems from the notion that milking the cow immediately after birth could deprive the calf of essential food, potentially harming or even killing it. This practice contributes to the delayed initiation of milking and, in turn, affects the milk supply available for sale.

The reluctance to milk the cow early and the cultural preference for sour milk highlight the need for targeted and culturally sensitive extension services (5, 23). Improving animal health management systems and providing education on efficient and sustainable milk production practices could address these challenges, encouraging farmers to optimize their milk production while meeting cultural and dietary preferences. It underscores the importance of integrating cultural practices into interventions to enhance their effectiveness in the context of the Western Province.

### 3.10 Sparsely located livestock population and markets

The study highlights a significant logistical challenge in Western Province related to the distance between communal grazing areas in the floodplains and the location of milk collection centers. While the optimal scenario is to have milk collection centers within a radius of at least 10 km from where animal production is taking place (24), the study revealed that they were located as far as 70 km away from some farmers in Western Province. This considerable distance poses several challenges, particularly in ensuring the quality of the collected milk for processors.

The remote location of milk collection centers makes transportation and timely collection of milk challenging. As a result, there may be difficulties in maintaining the freshness and quality of the milk during transportation over such long distances. This situation not only affects the processors’ ability to obtain good-quality milk but also impacts the overall efficiency and sustainability of the dairy value chain in the region.

Addressing this issue may involve strategic planning to establish additional collection centers near communal grazing areas or exploring innovative solutions for efficient transportation. Improving the accessibility of milk collection centers can contribute to the overall success of dairy development interventions in the Western Province. A key informant stated that:

*“Because of a dairy collection point, you need a radius of about 10 km at least; outside that, what you get is B-to C-grade, and usually the processor cannot get that quality because of the distances to the milk collection point.”*

The study emphasizes the challenge of sparsely populated and decentralized livestock in Western Province, where animals can move freely between districts along the Zambezi floodplain. This decentralized distribution makes it challenging to collect efficiently and pool milk from various strata of cattle in the region. The sparse location of milk collection centers further complicates matters, increasing the cost of transportation to the central processing plant.

For economic viability, establishing collection points becomes more feasible when a sufficient volume of milk offsets transportation costs (25). In the case of the Western Province, it’s suggested that a well-planned network of multiple milk collection centers, each producing a substantial volume of milk, could facilitate efficient transportation to the central processing plant. A similar strategy could be implemented in the Western Province by drawing parallels with successful models in other provinces, such as the Southern Province, where trucks collect milk from multiple collection centers in one trip.

For beef, transportation challenges are also evident, with some farmers and traders walking for 4 days to sell cattle to the nearest abattoir in Limulunga District. However, some initiatives, such as the establishment of loading points along key roads like the Mongu-Kalabo road, aim to facilitate cattle collection from various areas. Additionally, the Tapo area has been used as a collection point for milk and cattle, demonstrating the potential for strategically located collection points to streamline livestock value chains.

Addressing these challenges requires holistic interventions along the entire livestock value chain, including establishing strategically located collection points, efficient transportation networks, and collaborative efforts involving regional stakeholders.

### 3.11 Lack of knowledge and sensitization

The study highlighted a critical issue in Western Province related to a lack of knowledge about producing and handling milk among traditional dairy farmers. This knowledge gap has led to the production of low-quality milk in the region due to a deficiency in technical know-how. The cultural preference for highly sour milk further complicates the situation, necessitating training and sensitization programs for farmers to adapt to fresh milk handling practices.

Given the cultural background of the Western Province, where people are accustomed to handling highly sour milk, there is a need for targeted education and awareness campaigns. Farmers may not be familiar with the requirements and best practices for handling fresh milk, leading to challenges in maintaining its quality. The study noted that, due to this cultural background, farmers delivered milk to the collection center at various times, often 3–4 h after milking, resulting in the rejection of soured or fermented milk.

The rejection of sour or fermented milk at the collection center discourages farmers, potentially reducing their enthusiasm for milk production. To address this, interventions should focus on providing education and training programs that align with the cultural context of the region. This could include promoting best practices for milk handling, emphasizing the benefits of delivering fresh milk, and offering support to farmers to enhance the overall quality of milk production in Western Province.

### 3.12 Lack of transport

The study underscores the transportation challenges in Western Province, where the long distance to sparsely located milk collection centers requires reliable and specialized transportation of milk from the production sites in the Zambezi floodplain to the nearest collection points and processing plants. The unique sandy terrain of Western Province demands full-time four-wheel-drive transport, which further complicates the logistics and affects participation in markets.

The sandy terrain presents a notable hurdle, particularly for traditional cattle farmers who may find it economically impractical to travel long distances, whether by motorbike or bicycle, to supply relatively small volumes of milk to the milk collection center. In this context, the opportunity cost for delivering a small volume of milk becomes high, discouraging farmers from actively participating in the market.

This situation highlights the importance of considering both the distance to collection centers and the volumes of milk produced when designing transportation interventions. Effective solutions must consider the specific challenges the terrain poses and ensure that transportation methods are suitable for the conditions in Western Province. Addressing these transportation challenges is crucial for facilitating the efficient flow of milk from production sites to collection points and ultimately contributing to the overall success of dairy development interventions in the region. A focus group discussant had this to say:

*“To connect the whole story, transhumance systems have affected us, animals are very far away, poor genetics of our local breed leads to low milk volumes, low milk volumes which are far and need reliable transport, but again reliable transport like a motorbike is not economically viable due to high cost of fuel. That is why I say we have a lot of activity without productivity. I now have a bike, I can be bringing milk everyday here using a motorbike, but you know the liters I produce. Is it viable for me to ride a bike for the little volume? People can be bringing milk on bikes every, but will they really get more than the cost of fuel at the end of the month?”*

The study underscores that inadequate transportation infrastructure can easily impede farmers’ access to markets. Poor road conditions may increase transportation costs and difficulties getting cattle and milk to market centers.

### 3.13 Poor governance of dairy cooperatives

Poor governance systems of cooperatives in Western Province discourage members from joining or indeed continuing to supply milk. Cooperative members of the executive who believe they own the cooperative have a stronghold on cooperatives. They do not hold annual general meetings (AGM); some have been there for a long time, with some thinking that the cooperative was donated to them. The MCCs are run by an executive committee composed of the chairperson, vice chairperson, secretary, vice secretary, treasurer, vice treasurer, and a varying number of committee members. Apart from Mongu Dairy Cooperative, whose executive is composed of men, others had a mix of one or two women, with the rest being men. There is a tendency to select those who can communicate in English with a reasonable level of education. However, focus group discussants reported the absence of unity of purpose in all dairy cooperatives, as quoted below:

*“So again, the issue of working together—us here in the Western Province coming up with a cooperative to work together—is a problem compared to our friends in the Southern. We are very far from seeing the benefits of coming up with a group and working together; there is jealousy in there, and my friend will also buy this, so they have not seen the benefits of working together as a group…”*

In all the dairy cooperatives, membership has decreased from over 80 members to less than 20 members who are attempting to resuscitate the operations of MCCs. All dairy cooperatives in Western Province cannot meet overhead costs because they are not in production.

These governance challenges can undermine the cooperative’s effectiveness, discourage member participation, and hinder the overall success of regional dairy development initiatives. Addressing these issues requires implementing measures to ensure transparent and inclusive governance structures, promoting gender diversity, and fostering an environment encouraging active participation and input from all cooperative members.

### 3.14 The traditional governance system

The traditional governance system in Western Province follows a hierarchical structure from the village (Silalo) to the district Kuta and further to the Ngambela Kuta. This system is pivotal in mobilizing farmers and coordinating their participation, especially in supplying to collection points. The hierarchy, from the local Indunas at the village level to the Ngambela Kuta and the King, is highly respected and deemed critical for successful interventions in the livestock sector. The traditional governance system oversees communal grazing areas in the Zambezi floodplain, underscoring its central role in managing livestock-related resources. Any interventions in the province should acknowledge and integrate this traditional governance system for cultural appropriateness and effectiveness. A key informant stated that:

*“Indunas can affect participation in livestock markets, especially if they themselves are not involved. An intervention on livestock can come, but if the village of Induna is not an interested party, he can advise his subjects not to adopt market interventions…”*

The Barotse Royal Establishment (BRE) should enhance its on-the-ground structures and systems. Beyond presiding over civil disputes, the Kuta should actively engage in development discussions. It is recommended that the BRE make deliberate efforts to convene stakeholders, including the private sector, farmers, and public sector representatives. This collaborative approach aims to harmonize and coordinate regional livestock value chain development activities. By fostering partnerships and inclusivity, the BRE can be more proactive in driving holistic development initiatives within the livestock sector.

### 3.15 Lack of a holistic approach to value chain development

Livestock value chains in Western Province face underdevelopment characterized by weak linkages, poor coordination, and governance issues. This situation disadvantaged small-scale cattle farmers in terms of market bargaining power. Additionally, these farmers have limited access to affordable credit, hindering opportunities for improving production and adding value at the group or farm level. The absence of a sustainable and holistic approach to livestock value chain development from intervening entities has resulted in a lack of meaningful participation in livestock markets in Western Province. Addressing these challenges is crucial for fostering a more robust and inclusive livestock value chain in the region. A key informant had this to say:

*“The past interventions in Western Province missed out on people that can be used as models in livestock production. Interventions in Western Province tend to look at the value chain in terms of interests and only focus on an area they know best. Funders sometimes only look at an area they can manage well. However, if you know you are looking at the value chain holistically, from production inception to end-user, there could be so many players on the market. For instance, if you focus on the milking plant and do nothing about the production end, where will the milk come from? So you need to play a balanced game. You need to make sure that the production will feed the market. That, to me, is a very realistic approach rather than doing blanket appeasement of whatever and end up achieving nothing. So have it on record that the value chain must be looked at in total, and people to be empowered must be provided with the necessary skills to manage what we are promoting…”*

### 3.16 Lack of grading system in beef markets

The lack of a grading system for beef does not motivate the farmers to sell the best animals. This is because the one selling an almost dead animal (cull) gets the same price as the one selling a fattened animal in Western Province. This affects participation in livestock markets. There is a need to develop standards for grading meat not only in Western Province but also in Zambia so as to incentivize production. However, weak and lack of enforcement of standards in informal beef markets may affect grading systems. Meat from informal markets is sold at lower prices. The lower prices may threaten market competition with large processors, who may observe acceptable meat grading standards. A focus group discussant had this to say:

*“But if our animals were graded like this, from this weight to that weight, this would be the price. That one can motivate farmers, because, like I have seen across Namibia, that is what is happening: the animals are graded…”*

### 3.17 Uncompetitive pricing

The prices for livestock in Western Province have been very uncompetitive until recently, when a new player (anonomity) came onto the beef market. This development has increased the dressed weight from ZMK18.00 (about US$1) in 2019 to ZMK 35.00 (US$2.1) per kg dressed weight in 2020. The monopsony type of market in Western Province has played a significant role in discouraging participation in beef markets. A monopsony is a market structure in which a single buyer substantially controls the market as the major purchaser of goods and services offered by many producers or sellers, meaning that a buyer has all the freedom to set prices and thrive on trends in producer financial needs. For a long time, Western Province has had seven processors, with one processor dominating with more than 50% of the market share until the eighth processor came on the market and made prices competitive. The new entrant in the beef market seems to have brought competition to the beef industry in Western Province, although they cite tough competition as one of the challenges they face. The key informant stated that:

*The first one is competition; we have a lot of abattoirs and beef traders in the province. I think that is the major challenge. In fact, the majority of those people who could supply those animals here are traders rather than those who are getting them on their own.*

Fluctuations in market prices and demand can create uncertainty for small-scale farmers in an uncompetitive market. Limited understanding of market dynamics may lead to suboptimal timing for selling cattle and ultimately lower the participation of smallholder farmers in the market.

### 3.18 Lack of dairy markets (dairy processor)

The closure of the milk processing plant in Mongu has had a discouraging impact on participation in dairy markets in Western Province. The plant’s presence previously stimulated production, with farmers planning to acquire dairy breeds to increase volumes. However, the closure has disrupted these gains, creating a setback for dairy farmers in the region. The absence of the processing plant has likely affected the motivation for increased dairy production and market participation. Efforts to address the challenges faced by the processing plant or explore alternative solutions could contribute to revitalizing the dairy market in Western Province. A key informant stated that:

*“Now the only thing that can cause this to happen is to have a reliable market, because if there is a market that will continuously buy milk from the farmers, then they can invest in improved animal health management because they know that they will get the returns. So if there is an assured market, farmers will make the investment, but if there is no assured market, farmers will be afraid to make these investments. So for me, what is required is a continuous market to allow farmers to say, Okay, even if I make this investment, I will get it back…”*

The study revealed that limited access to well-functioning markets may lead to reduced opportunities for small-scale farmers to sell their milk and also shows that proximity to markets significantly affects farmers’ ability to sell their milk in a quest to attain food and income security.

### 3.19 Continuous reduction in cattle population for the past 5 years

The cattle population in Western Province has experienced a notable reduction over the past 5 years, declining from 711,070 in 2015 to 450,949 in 2018 and reaching 480,500 in 2020 (3). Several factors contribute to this decline, including prevalent diseases such as anthrax, CBPP, and occasional outbreaks of foot-and-mouth disease (FMD) (11, 26). The test and slaughter policy for CBPP control has led to cattle depopulation, impacting population growth rates (11). Liver fluke infestations, with over 90% of bovine livers condemned, have further affected animal production, reproduction, and participation in formal off-take markets (27).

Cattle sales play a significant role in the province’s economy, especially since there are limited alternative agricultural activities. Unlike provinces with extensive crop production, Western Province relies heavily on cattle sales as a primary livelihood source. The region has faced challenges such as drought, affecting rice production and fishing, exacerbating the reliance on cattle sales. Low cattle prices over the past 5 years, averaging around ZMK 10–12 (US$0.55) per dressed weight, have compelled farmers to sell more animals to meet financial needs, contributing to the reduction in the cattle population (10). Although recent price increases to ZMK35 (US$2.1) per dressed weight offer some relief, challenges like inflation rates continue to impact farmers’ decisions to sell fewer animals for the same financial requirements.

## 4 Conclusion

Given the limitations of seasonal and flood-prone soils for crop farming, this study underscores the vital role of livestock farming in Western Province. Despite the significance of livestock, the region faces challenges characterized by low productivity, limited participation in dairy and beef markets by small-scale farmers, and restricted access to formal off-take markets. The identified factors limiting the farmers’ engagement in livestock markets encompass the absence of commercial farmers, lack of improved dairy breeds, inadequate business mindset and skills, poor road infrastructure, insufficient supportive infrastructure, cultural practices and beliefs, scattered livestock population and markets, insufficient knowledge and sensitization, lack of transport, underdeveloped value chains, ineffective governance in dairy cooperatives, absence of a grading system in beef markets, and uncompetitive pricing. These factors also hinder private sector investment in Western Province. The study emphasizes the need to address these multifaceted challenges for the success of market interventions and livestock development programs in the region.

Understanding and addressing these factors can contribute to developing targeted interventions and policies to improve small-scale cattle farmers’ market participation in the Western Province of Zambia. This may involve collaborative efforts from government agencies, NGOs, and other stakeholders to create an enabling environment for sustainable and profitable livestock farming.

## Data Availability

The original contributions presented in the study are included in the article/supplementary material, further inquiries can be directed to the corresponding author.

## Footnotes

^1^ http://data.sasscal.org/metadata/view.php?view=geodata&id=5722
